# The Obstetrical Remembrancer, or Denman’s Aphorisms, &c.

**Published:** 1848

**Authors:** 


ARTICLE VI.

*The Obstetrical Remembrancer, or Denman's Aphorisms on Natural and Difficult Parturition; the application of instruments, &c.* Augmented by MICHAEL RYAN, M.D. First American from the ninth London edition, with additions by THOMAS F. COCK, M.D., Visiting Physician to the New York Lying-in Asylum. 18mo. pp. 264. New York: S. S. & W. Wood, 1848. (From the Publishers—for sale by Joseph Keene, Jr., Chicago.)

A concise collection of the most important facts in relation to obstetrics, and rules for the direction of the practitioner is here presented to the profession. The name of Denman, may give the idea of old and obsolete notions; but an examination of the work, will show that it is erroneous. An examination of the medical fathers occasionally, is not only interesting and instructing, but serves to remind us that most of the truths we have are not of modern discovery. [E.

